# Fibrinogen-to-albumin ratio percentage: An independent predictor of disease severity and prognosis in anti-N-methyl-D-aspartate receptor encephalitis

**DOI:** 10.3389/fneur.2023.1083752

**Published:** 2023-02-24

**Authors:** Juan Du, Yingzhe Shao, Yajun Song, Kaixin Wang, Xuan Yang, Yanfei Li, Yaobing Yao, Zhe Gong, Yanjie Jia

**Affiliations:** Department of Neurology, The First Affiliated Hospital of Zhengzhou University, Zhengzhou, China

**Keywords:** fibrinogen, albumin, fibrinogen-to-albumin ratio percentage, anti-NMDAR encephalitis, inflammation, prognosis, disease severity

## Abstract

**Purpose:**

This retrospective study aimed to investigate the relationship between fibrinogen-to-albumin ratio percentage (FARP) and disease severity and prognosis in patients with anti-N-methyl-D-aspartate receptor (anti-NMDAR) encephalitis.

**Methods:**

Medical records and clinical characteristics from 181 patients with anti-NMDAR encephalitis were included. The modified Rankin Scale (mRS) was used to analyze disease severity and prognosis at admission and discharge, and correlations between FARP, disease severity, and prognosis were analyzed. Receiver operating characteristic (ROC) curves were used to evaluate the efficiency of FARP in assessing disease severity and prognosis.

**Results:**

Compared to the control group, patients with anti-NMDAR encephalitis had higher fibrinogen (Fib) levels (*P* < 0.001), neutrophil counts (*P* < 0.001), and FARP levels (*P* < 0.001) but had lower albumin levels (P = 0.003). The enrolled patients were divided into mild-to-moderate and severe groups according to their mRS scores both at admission and discharge. FARP levels were significantly elevated in the severe group compared to the mild-to-moderate group among patients with anti-NMDAR encephalitis both at admission and discharge (admission 6.0 vs. 7.40, *P* < 0.001; discharge 6.43 vs. 8.18, P<0.001). Indeed, the mRS scores at admission (56 vs. 26%, *P* < 0.001) and discharge (26 vs. 11%, *P* = 0.006) in the high FARP group were significantly higher than those in the low FARP group. Furthermore, FARP was positively correlated with the mRS scores at admission (*r* = 0.383, *P* < 0.001) and discharge (r =0.312, P < 0.001). In the multivariate analysis, FARP was significantly associated with disease severity (odds ratio [OR] = 1.416, 95% confidence interval [CI] = 1.117-1.795, *P* = 0.004) and prognosis (OR = 1.252, 95% CI = 1.010-1.552, *P* = 0.040). FARP-based ROC curves predicted disease severity, with a sensitivity of 0.756, a specificity of 0.626, and an area under the ROC curve of 0.722 (95% CI = 0.648–0.796, *P* < 0.001^*^). The ROC curve predicted the disease prognosis with a sensitivity of 0.703, a specificity of 0.667, and an area under the ROC curve of 0.723 (95% CI = 0.629–0.817, *P* < 0.001^*^).

**Conclusion:**

Our results indicate that FARP is a novel predictive marker for disease severity and prognosis of anti-NMDAR encephalitis.

## 1. Introduction

Anti-N-methyl-D-aspartate receptor (anti-NMDAR) encephalitis is a disease characterized by a series of neurological and psychiatric symptoms. It is mainly triggered by an autoimmune process mediated by specific antibodies against the GluN1 subunit of the NMDAR in the cerebrospinal fluid (CSF) (1). It was first discovered in 2007 in female patients with ovarian teratoma (2); it is a disorder that presents with a severe neuropsychiatric manifestation characterized by psychiatric manifestations, seizures, movement disorders, disturbance of consciousness, autonomic nerve disorders, or insufficiency of ventilation (2, 3). In general, patients with anti-NMDAR encephalitis maintain a good clinical prognosis; however, it can also lead to intractable epilepsy, serious mental disorders, and even death in some patients (4). Mechanically, a variety of immune cells, including B cells, Th17 cells, and cytokines such as IL-1β, IL-6, IL-7, and IL-23, are involved in the pathophysiology of anti-NMDAR encephalitis and play a crucial role in the occurrence and development of the disease (5, 6). At present, a set of clinical features and NMDAR antibodies in the CSF and serum are the main criteria for the diagnosis of anti-NMDAR encephalitis. The identification of biological indicators that can predict disease severity and prognosis is the focus of current research.

The blood–brain barrier (BBB) is a natural barrier that prevents pathogens, neurotoxic plasma components, and blood cells from entering the brain and maintains the brain's integrity under normal circumstances. Many central nervous system (CNS) diseases are related to the entry of toxic metabolites into the CNS after the damage of the BBB, resulting in neuronal dysfunction and cell death and leading, in turn, to diseases such as stroke, multiple sclerosis, epilepsy, Alzheimer's disease, and Parkinson's disease (7). The current view is that anti-NMDAR encephalitis is probably triggered by viruses and tumors, and this is caused by disruption of the BBB through autoimmune inflammatory responses. In fact, NMDAR antibodies, which can directly bind to NMDAR sites, as well as lymphocytes and other immunocytes, can enter the CNS through the disrupted BBB (8).

Fibrinogen (Fib) is a key molecular player in the coagulation mechanism and has a pro-inflammatory role in stroke, traumatic brain injury, spinal cord injury, multiple sclerosis, Alzheimer's disease, vascular wall disease, rheumatic immune diseases, and several types of cancer (9–11). Coagulation mechanisms are intimately linked to the immune system. Increasing evidence shows that abnormal coagulation function is related to autoimmune diseases, and damage and inflammation can be caused by high immune-cell activity and BBB permeability (12). Fib has been shown to modulate the levels of C-reactive protein (CRP) during inflammatory processes (13), and Zhang et al. found that in the non-remission group of patients with anti-NMDAR encephalitis, Fib levels were higher than those in the control group. However, whether a causative association exists between Fib and anti-NMDAR encephalitis remains unclear (14).

Albumin (Alb) is an objective indicator of nutritional status (15). Previous studies have reported that Alb diffusion into the CNS occurs before cellular inflammation and clinical manifestations of CNS diseases. Moreover, high Alb levels are predictors of a favorable response to early immunotherapy in autoimmune encephalitis (16).

Fib and Alb play important roles in inflammation. The Fib/Alb ratio percentage (FARP) has been shown to predict disease severity and prognosis in various inflammatory, metabolic, and neoplastic diseases. However, concerning the most common autoimmune encephalitis, the anti-NMDAR type, no research has so far reported whether FARP can act as a predictor of disease severity and prognosis. Thus, in the present study, we determined the levels of FARP, Fib, and Alb and analyzed the capacity of FARP in predicting the severity of neurological impairment and prognosis of anti-NMDAR encephalitis.

## 2. Methods

### 2.1. Patients

**Figure 1:**
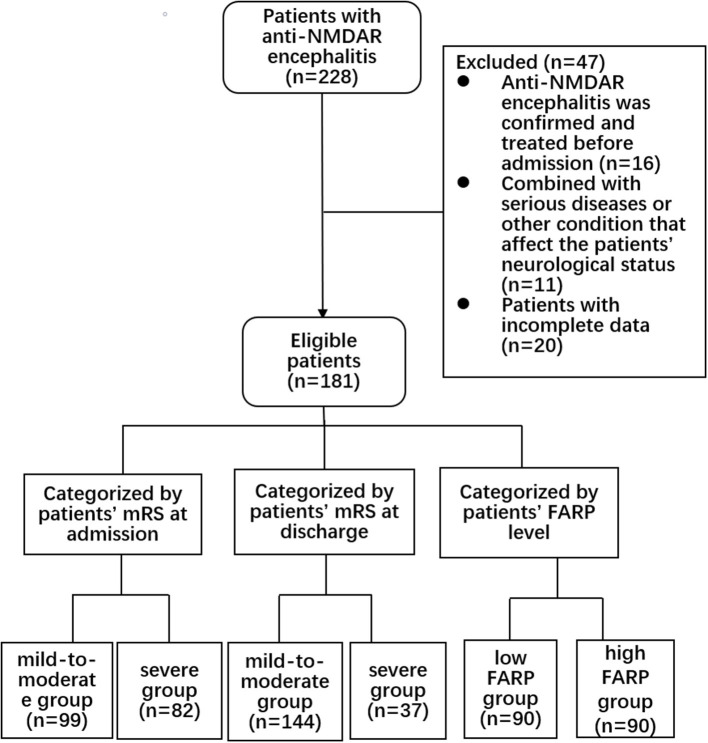
Flow chart showing the patient screening process.

This retrospective cohort study was approved by the Zhengzhou University Ethics Committee (2019-KY-018). All the patients submitted written informed consent for participation. We collected the clinical data of 228 patients in the Department of Neurology of the First Affiliated Hospital of Zhengzhou University from April 2014 to April 2021. All patients met the international diagnostic criteria of anti-NMDAR encephalitis (17), with at least one of the following symptoms: (1) psychiatric disturbance, seizures, abnormal movement, speech disorder, consciousness declination, and autonomic dysfunction/central hypoventilation; (2) CSF positive for anti-NMDAR antibodies; and (3) free of other diseases. The exclusion criteria were as follows: (1) anti-NMDAR encephalitis previously diagnosed and treated with corticosteroids, intravenous immunoglobulin, immunosuppressants, plasma exchange, and other immunotherapies in other medical institutions before admission (*n* = 16); (2) with another comorbid serious disease, such as tumor, recurrent serious infection, recent use of anticoagulants, or other conditions affecting the nervous system (*n* = 11 patients); and (3) patients with incomplete data (*n* = 20). The screening process is shown in Figure 1.

### 2.2. Data collection

The data collected in this retrospective study were obtained through the hospital's electronic medical record system and included baseline information (sex, age, and past medical history), clinic symptoms (main onset phenotype and state of consciousness), laboratory examinations (blood cell count, total protein, Alb, globulin, coagulation function index, CRP, and FARP), electroencephalogram (EEG), magnetic resonance imaging (MRI), and treatment options. Fasting venous blood was collected from the patients early in the morning after admission. All blood samples acquired for routine blood tests and biomarker identification were analyzed in the biochemistry laboratory of the First Affiliated Hospital of Zhengzhou University. All tests were performed according to the manufacturer's instructions and relevant guidelines, and the inspectors were blinded to all clinical information.

### 2.3. Clinical assessment

The neurological function of patients with anti-NMDAR encephalitis was assessed using the modified Rankin Scale (mRS) (18). The levels of this scale are as follows: no symptoms (score 0); no obvious dysfunction can complete all daily activities and duties despite symptoms (score 1); mild disability and cannot complete all previous affairs but able to accomplish personal routine without assistance (score 2); with a moderate disability require some help but did not need assistance when walking (score 3); severely disabled, unable to walk without assistance, and unable to look after their own physical needs (score 4); severe disability, completely bedridden, incontinent, and in need of continuous care (score 5); and death (score 6). Patients were divided into a mild-to-moderate group with mRS scores of ≤ 3 and a severe group with mRS scores of >3. The initial mRS at admission was used as an index to evaluate disease severity, and the final mRS at discharge was used as an index to evaluate prognosis.

Statistical analysis was performed using the SPSS software package (version 25.0; IBM, Armonk, NY, USA). Continuous data obtained in this study with normal distribution were expressed as mean ± standard deviation (x ± s). Continuous data with a non-normal distribution were expressed as median with interquartile range (IQR). The differences between the two groups were analyzed using Student's *t*-test and the Mann–Whitney U-test for normally and non-normally distributed data, respectively. Categorical variables were presented as frequency (percentage, %), and the chi-squared tests were used for comparison between the groups. Spearman's rank analysis or Pearson's correlation analysis, as appropriate, was used to assess the correlations between the profiles. In the logistic regression analysis, the initial mRS score and the final mRS score represent disease severity and prognosis, respectively, which were taken as dependent variables. Univariate logistic regression models were used to evaluate whether FARP or other covariates had an independent influence on the severity and prognosis of the disease (*P* < 0.1), and multivariate logistic regression models were used to analyze the independent influence of FARP on the severity and prognosis of the disease. The results were expressed as odds ratios (OR) and 95% confidence intervals (95% CI). The receiver operating characteristic (ROC) curve was plotted to assess the predictive ability of FARP for disease severity and prognosis and to determine the criterion value of FARP for patients with anti-NMDAR encephalitis. A two-tailed *P-*value of < 0.05 was considered statistically significant.

## 3. Results

### 3.1. Clinical and demographic characteristics

Ninety-one female patients (50.3%) aged 27 on average (18–42) were included in the anti-NMDAR encephalitis group (*n* = 181). In the control group (*n* = 198), 107 female patients (54.0%) aged 29.5 years on average (17.8–46) were included. There were no remarkable differences in age and gender between the two groups (*P* > 0.05). The platelet count, basophil count, and incidence of diabetes and hypertension did not differ significantly between the two groups (*P* > 0.05). We observed significant intergroup differences in white blood cell (WBC) count, red blood cell (RBC) count, hemoglobin, neutrophil count, monocytes count, lymphocytes count, eosinophil count, prothrombin time (PT), activated partial thromboplastin time (APTT), Fib, thrombin time (TT), D-Dimer, albumin, and FARP (*P* < 0.05) (Table 1).

**Table 1:** Clinical characteristics of the patients with anti-NMDAR encephalitis and healthy controls.

	**Anti-NMDAR encephalitis (*n* = 181)**	**Normal contral group ( *n* = 198)**	***P***
**Demographic data**			
Age at onset, years, median (IQR)	27 (18-42)	29.5 (17.8–46)	0.169
Gender, female, *n* (%)	91 (50.3)	107 (54.0)	0.464
**Clinical features at admission**			
Diabetes, *n* (%)	4 (2.2)	5 (2.5)	1.000
Hypertension, *n* (%)	16 (8.8)	21 (10.6)	0.563
**Laboratory test results, median (IQR)**			
WBC, median (IQR), × 10^9^/L	6.1 (4.96–7.09)	9.0 (6.7–11.9)	< 0.001^*^
RBC, mean (SD), × 10^12^/L	4.29 (4.01–4.70)	4.47 (4.16–4.76)	0.039^*^
Hemoglobin, median (IQR), g/L	131 (119–142)	134 (124–145)	0.023^*^
Platelet, median (IQR), × 10^9^/L	242 (197.25–307.75)	235 (207–270)	0.160
Neutrophil, median (IQR), × 10^9^/L	6.79 (4.40–9.89)	3.34 (2.63–4.16)	< 0.001^*^
Monocytes, median (IQR), × 10^9^/L	0.57 (0.43-0.76)	0.42 (0.34–0.49)	< 0.001^*^
Lymphocytes, median (IQR), × 10^9^/L	1.51 (1.09–2.14)	1.95 (1.61–2.37)	< 0.001^*^
eosinophil, median (IQR), × 10^9^/L	0.04 (0.01–0.09)	0.11 (0.06–0.18)	< 0.001^*^
basophil, median (IQR), × 10^9^/L	0.03 (0.01–0.04)	0.03 (0.02–0.04)	0.135
PT, median (IQR), s	11.0 (10.5–11.7)	10.7 (10.1–11.2)	< 0.001^*^
APTT, median (IQR), s	28.2 (25.9–31.3)	30.4 (28.4–32.9)	< 0.001^*^
FIB, median (IQR), g/L	2.85 (2.41–3.41)	2.51 (2.16–2.96)	< 0.001^*^
TT, median (IQR), s	14.4 (13.4–15.8)	15.65 (14.6–17.13)	< 0.001^*^
D-Dimer, median (IQR), mg/L	0.18 (0.08–0.48)	0.07 (0.04–0.19)	< 0.001^*^
albumin, mean (SD), g/L	42.7 (39.1–45.8)	43.5 (41.2–46.6)	0.003^*^
FARP, median (IQR)	6.85 (5.42–8.18)	5.88 (4.87–6.84)	< 0.001^*^

mRS, modified Rankin scale; IQR, interquartile range; ICU, intense care unit; RBC, red blood cell; WBC, white blood cell; PT, prothrombin time; APTT, activated partial thromboplastin time; FIB, fibrinogen; TT, thrombin time; FARP, fibrinogen albumin ratio percentage.

^*^P < 0.05.

The inclusion and exclusion criteria are shown in Figure 1. In the anti-NMDAR encephalitis group, 106 patients (58.6%) were admitted to the intensive care unit (ICU) for treatment, and the time from admission to discharge was 26 (17.00–40.50) days. Eleven patients (6.1%) had viral encephalitis before onset, and 91 patients (50.3%) had consciousness disorders. In the whole cohort, psychiatric disturbances and seizures were the main clinical symptoms; 114 patients (63.0%) had psychiatric disturbances, and 110 patients (60.8%) had seizures.

To determine the factors affecting the severity of the disease, we divided the patients into mild-to-moderate and severe groups based on whether the mRS score was ≤ 3 or >3 at admission. As shown in Table 2, we compared their clinical and demographic characteristics. We observed no remarkable differences between the groups concerning sex, age, time from admission to discharge, or prevalence of diabetes, hypertension, viral encephalitis, seizures, sleep disorders, memory impairment, movement disorders, speech dysfunctions, EEG, or MRI abnormalities (*P* > 0.05). We observed significant intergroup differences in ICU admission, consciousness disorders, psychiatric disturbance, autonomic nerve dysfunction, neutrophils, FARP, Fib, CRP, lymphocytes, eosinophils, basophils, D-dimer, and Alb levels (*P* < 0.05). The levels of neutrophils (*P* < 0.001), FARP, Fib, and CRP were noticeably higher in the severe group than in the mild-to-moderate group.

**Table 2:** Comparison of clinical data of anti-NMDAR encephalitis with different severity at admission.

	**Total (*n* = 181)**	**I–mrs ≤ 3 (*n* = 99)**	**mrs >3 (*n* = 82)**	***P***
**Demographic data**				
Age at onset, years, median (IQR)	27 (18.0–42.0)	25 (19.0–42.0)	27 (17.0–41.5)	0.752
Gender, female, *n* (%)	91 (50.3)	45 (45.5)	46 (56.1)	0.154
**Clinical features at admission**				
Time from admission to discharge, median (IQR)	26 (17.0–40.5)	24 (18.0–36.0)	29 (16.8–46.3)	0.126
ICU admission, *n* (%)	106 (58.6)	34 (34.3)	72 (87.8)	< 0.001^*^
diabetes, *n* (%)	4 (2.2)	2 (2.0)	2 (2.4)	1
hypertension, *n* (%)	16 (8.8)	9 (9.1)	7 (8.5)	0.896
Viral encephalitis, *n* (%)	11 (6.1)	8 (8.2)	3 (3.7)	0.209
Consciousness disorders, *n* (%)	91 (50.3)	22 (22.2)	69 (84.1)	< 0.001^*^
Psychiatric behavior, *n* (%)	114 (63.0)	51 (51.5)	63 (76.8)	< 0.001^*^
Seizures, *n* (%)	110 (60.8)	54 (54.5)	56 (68.3)	0.059
Sleep disorders, *n* (%)	60 (33.1)	31 (31.3)	29 (35.4)	0.564
Memory disorders, *n* (%)	84 (46.4)	43 (43.4)	41 (50.0)	0.378
Movement disorders, *n* (%)	31 (17.1)	13 (13.1)	18 (22.0)	0.117
Speech dysfunction, *n* (%)	57 (31.5)	31 (31.3)	26 (31.7)	0.955
Autonomic dysfunction, *n* (%)	40 (22.1)	11 (11.1)	29 (35.4)	< 0.001^*^
**Laboratory test results, median (IQR)**				
RBC, median (IQR), × 10^12^/L	4.29 (4.01–4.70)	4.44 (4.03–4.77)	4.22 (3.94–4.63)	0.218
platelet, median (IQR), × 10^9^/L	242.00 (197.25–307.75)	246.50 (201.75–308.25)	240.00 (181.75–299.00)	0.351
Neutrophil, median (IQR), × 10^9^/L	6.79 (4.40–9.89)	5.20 (3.66–7.43)	8.97 (5.69–11.45)	< 0.001^*^
Monocytes, median (IQR), × 10^9^/L	0.57 (0.43–0.76)	0.52 (0.43–0.72)	0.61 (0.46–0.86)	0.097
Lymphocytes, median (IQR), × 10^9^/L	1.51 (1.09–2.14)	1.72 (1.40–2.28)	1.29 (0.76–2.72)	< 0.001^*^
eosinophil, median (IQR), × 10^9^/L	0.04 (0.01–0.09)	0.06 (0.02–0.12)	0.02 (0.00–0.07)	< 0.001^*^
basophil, median (IQR), × 10^9^/L	0.03 (0.01–0.04)	0.03 (0.02–0.04)	0.02 (0.01–0.04)	0.046^*^
FARP, median (IQR)	6.85 (5.42–8.18)	6.00 (5.02–7.52)	7.40 (6.49–9.49)	< 0.001^*^
APTT, median (IQR), s	28.2 (25.9–31.3)	29.3 (26.9–32.0)	27.5 (25.6–30.8)	0.046
FIB, median (IQR), g/L	2.85 (2.41–3.41)	2.63 (2.30–3.35)	2.97 (2.68–3.62)	< 0.001
TT, median (IQR), s	14.40 (13.40–15.80)	14.60 (13.30–16.00)	14.30 (13.35–15.45)	0.037
D–Dimer, median (IQR), mg/L	0.18 (0.08–0.48)	0.95 (0.06–0.25)	0.66 (0.31–1.97)	< 0.001^*^
albumin, median (IQR), g/L	42.70 (39.10–45.80)	44.70 (41.63–46.38)	40.30 (38.05–44.30)	< 0.001^*^
CRP, median (IQR), mg/L	2.20 (1.00–8.16)	1.50 (0.70–4.56)	5.18 (1.19–14.50)	0.001^*^
**Imaging examination**				
abnormal MRI, *n* (%)	73 (46.2)	43 (46.7)	30 (45.5)	0.873
abnormal EEG, *n* (%)	24 (68.6)	17 (70.8)	7 (63.6)	0.973

mRS, modified Rankin scale; IQR, interquartile range; ICU, intense care unit; RBC, red blood cell; FARP, fibrinogen albumin ratio percentage; APTT, activated partial thromboplastin time; FIB, fibrinogen; TT, thrombin time; CRP, C-reactive protein; MRI, magnetic resonance imaging; EEG, electroencephalograph.

^*^P < 0.05.

The mild-to-moderate and severe groups, based on whether the mRS score was ≤ 3 or >3 at discharge, were compared to determine whether any of the studied factors affected disease prognosis. As shown in Table 3, no significant differences were observed between the two groups regarding age, sex, time from admission to discharge, diabetes, hypertension, viral encephalitis, epilepsy, sleep disorders, memory impairment, movement dysfunction, speech disorders, red blood well as red blood cells (RBC), platelet count (PLT), monocytes, lymphocytes, basophils, activated partial thromboplastin time (APTT), thrombin time (TT), Alb, MRI, EEG, first- and second-line treatment, long-term immunotherapy, relapse, and other indicators (*P* > 0.05). We observed remarkable differences between the two groups at ICU admission, consciousness dysfunction, psychiatric disturbance, autonomic nerve dysfunction, neutrophils, eosinophils, FARP, Fib, D-dimer, and CRP (*P* < 0.05).

**Table 3:** Comparison of clinical data of anti-NMDAR encephalitis with different severity at discharge.

	**Total ( *n* = 181)**	**O–mrs ≤ 3 ( *n* = 144)**	**O–mrs>3 (*n* = 37)**	***P***
**Demographic data**				
Age at onset, years, median (IQR)	27 (18.0–42.0)	25 (17.3–40.8)	30 (22.0–45.5)	0.113
Gender, female, *n* (%)	91 (50.3)	71 (49.3)	20 (54.1)	0.606
**Clinical features from admission to discharge**				
Time from admission to discharge, median (IQR)	26 (17.0–40.5)	26 (18.0–39.8)	24 (13.5–50.0)	0.588
ICU admission, *n* (%)	106 (58.6)	71 (49.3)	35 (94.6)	< 0.001^*^
Diabetes, *n* (%)	4 (2.2)	4 (2.8)	0 (0.0)	0.583
Hypertension, *n* (%)	16 (8.8)	12 (8.3)	4 (10.8)	0.636
Viral encephalitis, *n* (%)	11 (6.1)	8 (5.6)	3 (8.1)	0.569
Consciousness disorders, *n* (%)	91 (50.3)	61 (42.4)	30 (81.1)	< 0.001^*^
Clinical phenotype at onset, *n* (%)				
Psychiatric behavior, *n* (%)	114 (63.0)	85 (59.0)	29 (78.4)	0.030^*^
seizures, *n* (%)	110 (60.8)	84 (58.3)	26 (70.3)	0.185
Sleep disorders, *n* (%)	60 (33.1)	44 (30.6)	16 (43.2)	0.144
Memory disorders, *n* (%)	84 (46.4)	65 (45.1)	19 (51.4)	0.499
Movement disorders, *n* (%)	31 (17.1)	24 (16.7)	7 (18.9)	0.746
Speech dysfunction, *n* (%)	57 (31.5)	44 (30.6)	13 (35.1)	0.593
Autonomic dysfunction, *n* (%)	40 (22.1)	27 (18.8)	13 (35.1)	0.032^*^
**Laboratory test results, median (IQR)**				
RBC, median (IQR) , × 10^12^/L	4.29 (4.01–4.70)	4.34 (4.03–4.73)	4.15 (3.84–4.63)	0.395
platelet, median (IQR), × 10^9^/L	242.00 (197.25–307.75)	242.00 (197.00–308.00)	242.00 (193.00–308.50)	0.958
Neutrophil, median (IQR), × 10^9^/L	6.79 (4.40–9.89)	6.15 (4.32–8.87)	9.21 (5.25–11.21)	0.002^*^
Monocytes, median (IQR), × 10^9^/L	0.57 (0.43–0.76)	0.57 (0.43–0.76)	0.64 (0.47–0.86)	0.466
Lymphocytes, median (IQR), × 10^9^/L	1.51 (1.09–2.14)	1.50 (1.10–2.10)	1.47 (0.90–2.17)	0.296
eosinophil, median (IQR), × 10^9^/L	0.04 (0.01–0.09)	0.04 (0.01–0.09)	0.02 (0.00–0.07)	0.023^*^
basophil, median (IQR), × 10^9^/L	0.03 (0.01–0.04)	0.03 (0.01–0.04)	0.03 (0.01–0.04)	0.296
FARP, median (IQR)	6.85 (5.42–8.18)	6.43 (5.29–7.71)	8.18 (6.59–9.94)	< 0.001^*^
APTT, median (IQR), s	28.20 (25.90–31.30)	28.50 (25.90–31.50)	28.20 (25.70–30.90)	0.56
FIB, median (IQR), g/L	2.85 (2.41–3.41)	2.82 (2.35–3.35)	3.12 (2.68–3.81)	< 0.001^*^
TT, median (IQR), s	14.40 (13.40–15.80)	14.40 (13.40–15.90)	14.40 (12.60–15.50)	0.101
D–Dimer, median (IQR), mg/L	0.18 (0.08–0.48)	0.14 (0.07–0.39)	0.34 (0.12–0.59)	0.002^*^
Albumin, median (IQR), g/L	42.70 (39.10–45.80)	43.00 (40.00–46.00)	40.60 (38.60–45.00)	0.050
CRP, median (IQR), mg/L	2.20 (1.00–8.16)	1.53 (0.85–7.61)	5.42 (1.33–22.52)	0.010^*^
**Imaging examination**				
abnormal MRI, *n* (%)	73 (46.2)	63 (48.8)	10 (34.5)	0.161
abnormal EEG, *n* (%)	24 (68.6)	22 (68.8)	2 (66.7)	1.000
**Treatment**				
First line immunotherapies	174 (97.8)	139 (98.6)	35 (94.6)	0.191
Second line immunotherapies, *n* (%)	9 (5.0)	6 (4.2)	3 (8.1)	0.576
Long term immunotherapies, *n* (%)	20 (11.0)	16 (11.1)	4 (10.8)	1.000
Relapse, *n* (%)	24 (13.3)	19 (13.2)	5 (13.5)	1.000

mRS, modified Rankin scale; IQR, interquartile range; ICU, intense care unit; RBC, red blood cell; FARP, fibrinogen albumin ratio percentage; APTT, activated partial thromboplastin time; FIB, fibrinogen; TT, thrombin time; CRP, C-reactive protein; MRI, magnetic resonance imaging; EEG, electroencephalograph.

^*^P < 0.05.

To better evaluate the relevance of FARP to disease severity and prognosis, we divided patients into a low FARP group (FARP ≤ 6.85, *n* = 90) and a high FARP group (FARP >6.85, *n* = 91) by the median FARP (Table 4). We observed no significant differences between the groups in sex, time from admission to discharge, psychiatric disturbance, seizures, sleep disorders, memory impairment, movement dysfunction, RBC, PLT, monocytes, eosinophils, basophils, APTT, serum NMDAR antibodies, EEG, first- and second-line treatment, long-term immunotherapy, or relapse (*P* > 0.05). Moreover, we observed noticeable differences between the groups in age, ICU admission, hypertension, viral encephalitis, consciousness dysfunction, neutrophils, lymphocytes, Fib, TT, D-dimer, Alb, CRP, the initial mRS, the finial mRS, and MRI findings (*P* < 0.05).

**Table 4:** Demographic and clinical data of patients with anti-NMDAR encephalitis in High FARP group (FARP ≤ 6.85) and Low FARP group (FARP >6.85).

	**Total (*n* = 181)**	**FARP ≤ 6.85 (*N* = 90)**	**FARP >6.85 (*N* = 91)**	***P***
**Demographic data**				
Age at onset, years, mean ± SD	25 (18-41)	21 (14.25–32)	31 (23.5–44)	< 0.001^*^
Gender, female, *n* (%)	91 (50.3)	49 (54.4)	42 (46.2)	0.265
**Clinical features from admission to discharge**				
Time from admission to discharge, median (IQR)	26 (18-43)	25.5 (17–38.75)	29 (18–47.5)	0.431
ICU admission, *n* (%)	106 (58.6)	46 (51.1)	60 (65.9)	0.043^*^
Diabetes, *n* (%)	4 (2.2)	1 (1.1)	3 (3.3)	0.621
Hypertension, *n* (%)	16 (8.8)	3 (3.3)	13 (14.3)	0.009^*^
Viral encephalitis, *n* (%)	11 (6.1)	9 (10.0)	2 (2.2)	0.029^*^
Consciousness disorders, *n* (%)	91 (50.3)	37 (41.1)	54 (59.3)	0.014^*^
Psychiatric behavior, *n* (%)	114 (63.0)	57 (63.3)	57 (62.6)	0.923
Seizures, *n* (%)	110 (60.8)	51 (56.7)	59 (64.8)	0.260
Sleep disorders, *n* (%)	60 (33.1)	33 (36.7)	27 (29.7)	0.317
Memory disorders, *n* (%)	84 (46.4)	39 (43.3)	45 (49.5)	0.409
Movement disorders, *n* (%)	31 (17.1)	16 (17.8)	15 (16.5)	0.817
Speech dysfunction, *n* (%)	57 (31.5)	29 (32.2)	28 (30.8)	0.833
Autonomic dysfunction, *n* (%)	40 (22.1)	16 (17.8)	24 (26.4)	0.163
**Laboratory test results, median (IQR)**				
RBC, median (IQR), × 10^12^/L	4.30 (4.03–4.72)	4.43 (4.08–4.75)	4.23 (3.98–4.70)	0.686
platelet, median (IQR), × 10^9^/L	242.00 (197.50–311.00)	249.00 (201.25–314.25)	240.00 (195.50–306.50)	0.709
Neutrophil, median (IQR), × 10^9^/L	6.87 (4.51–9.90)	5.55 (3.86–8.16)	7.90 (5.20–10.90)	< 0.001^*^
Monocytes, median (IQR), × 10^9^/L	0.58 (0.43–0.77)	0.56 (0.41–0.77)	0.58 (0.48–0.76)	0.411
Lymphocytes, median (IQR), × 10^9^/L	1.50 (1.07–2.10)	1.56 (1.23–2.31)	1.45 (0.79–2.00)	0.015^*^
eosinophil, median (IQR), × 10^9^/L	0.04 (0.01–0.08)	0.04 (0.01–0.07)	0.04 (0.01–0.09)	0.760
basophil, median (IQR), × 10^9^/L	0.03 (0.01–0.04)	0.03 (0.01–0.04)	0.03 (0.01–0.04)	0.035
APTT, median (IQR), s	28.20 (25.90–31.35)	28.55 (26.60–31.18)	28.20 (25.65–31.45)	0.573
FIB, median (IQR), g/L	2.87 (2.45–3.41)	2.45 (2.09–2.65)	3.38 (3.00–3.90)	< 0.001^*^
TT, median (IQR), s	14.40 (13.30–15.70)	15.50 (14.20–16.38)	13.60 (12.85–14.70)	< 0.001^*^
D–Dimer, median (IQR), mg/L	0.18 (0.08–0.48)	0.10 (0.06–0.28)	0.26 (0.12–0.60)	< 0.001^*^
albumin, median (IQR), g/L	42.80 (38.95–45.90)	44.65 (41.50–46.58)	40.50 (38.00–44.40)	< 0.001^*^
CRP, median (IQR), mg/L	2.20 (1.00–8.16)	1.28 (0.59–3.08)	5.18 (1.50–13.50)	< 0.001^*^
Serum anti–NMDAR antibody, *n* (%)	70/114 (61.4)	34/52 (65.4)	36/62 (58.1)	0.424
mRS upon admission, *n* (%)	82 (45.3)	26 (28.9)	56 (61.5)	< 0.001^*^
mRS upon discharge, *n* (%)	37 (20.4)	11 (12.2)	26 (28.6)	0.006^*^
**Imaging examination**				
abnormal MRI, *n* (%)	72/158 (45.6)	30/81 (37.0)	42/77 (54.5)	0.027^*^
abnormal EEG, *n* (%)	24/35 (68.6)	14/20 (70.0)	10/15 (66.7)	1.000
**Treatment**				
First line immunotherapies	174 (96.1)	88 (97.8)	86 (94.5)	0.450
Second line immunotherapies, *n* (%)	9 (5.0)	5 (5.6)	4 (4.4)	0.986
Long term immunotherapies, *n* (%)	20 (11.0)	9 (10.0)	11 (12.1)	0.654
Relapse, *n* (%)	24 (13.3)	11 (12.2)	13 (14.3)	0.682

mRS, modified Rankin scale; IQR, interquartile range; ICU, intense care unit; RBC, red blood cell; FARP, fibrinogen albumin ratio percentage; APTT, activated partial thromboplastin time; FIB, fibrinogen; TT, thrombin time; CRP, C-reactive protein; MRI, magnetic resonance imaging; EEG, electroencephalograph.

^*^P < 0.05.

### 3.2. Correlation between FARP and anti-NMDAR encephalitis disease severity

Spearman's correlation analysis showed that the blood lymphocyte count (*r* = −0.297, *P* < 0.001), APTT (*r* = −0.149, *P* = 0.046), Fib (*r* = 0.295, *P* < 0.001), TT (*r* = −0.156, *P* = 0.037), and CRP (*r* = 0.279, *P* = 0.001) were significantly correlated with the initial mRS scores of patients with anti-NMDAR encephalitis; however, the correlation was weak. FARP (*r* = 0.383, *P* < 0.001), Alb (*r* = −0.330, *P* < 0.001), blood neutrophil count (*r* = 0.427, *P* < 0.001), and D-dimer level (*r* = 0.428, *P* < 0.001) were significantly correlated with the initial mRS scores. Among these, FARP was found to be positively associated with the initial mRS score. No correlation was observed between blood monocyte count level and disease severity (*P* > 0.05) (Table 5).

**Table 5:** Correlation analysis of clinical data and mRS scores.

	**I–mrs**		**O–mrs**	
	***r***	***P***	***r***	***p***
Neutrophil, × 10^9^/L	0.427	< 0.001^*^	0.235	0.001^*^
Lymphocytes, × 10^9^/L	−0.297	< 0.001^*^	−0.078	0.298
Monocytes, × 10^9^/L	0.124	0.097	0.055	0.467
FARP, %	0.383	< 0.001^*^	0.312	< 0.001^*^
APTT, s	−0.149	0.046^*^	−0.043	0.562
FIB, g/L	0.295	< 0.001^*^	0.271	< 0.001^*^
TT, s	−0.156	0.037^*^	−0.123	0.101
D–Dimer, mg/L	0.428	< 0.001^*^	0.229	0.002^*^
Albumin, g/L	−0.330	< 0.001^*^	−0.146	0.050
CRP, mg/L	0.279	0.001^*^	0.211	0.010^*^

mRS, modified Rankin scale; FARP, fibrinogen albumin ratio percentage; APTT, activated partial thromboplastin time; FIB, fibrinogen; TT, thrombin time; CRP, C-reactive protein.

^*^P < 0.05.

To investigate the potential factor affecting disease severity, we performed univariate logistic regression analysis, which showed that the FARP (OR = 1.558, 95% CI = 1.299–1.868, *P* < 0.001), neutrophils (OR = 1.159, 95% CI = 1.069–1.255, *P* < 0.001), D-Dimer (OR = 2.634, 95% CI = 1.308–5.303, *P* = 0.007), and CRP (OR = 1.031, 95% CI = 1.001–1.063, *P* = 0.044) were significantly correlated with disease severity, as shown in Table 6. TT (OR = 0.872, 95% CI = 0.741–1.025, *P* = 0.097) had a moderate effect on the initial mRS score. Moreover, we observed no significant correlation with the initial mRS scores for age, sex, hypertension, blood monocyte and lymphocyte counts, APTT, and initial mRS scores (*P* > 0.1). The multivariate model showed that FARP was an independent risk factor for disease severity (OR = 1.416, 95% CI = 1.117–1.795, *P* = 0.004).

**Table 6:** Binary logistic regression of disease severity in patients with anti-NMDAR encephalitis.

	**Univariable analysis**		**Multivariable analysis**	
	**OR (95%CI)**	***P***	**OR (95%CI)**	***P***
Age at onset	1.006 (0.987–1.025)	0.544		
Gander, female	0.652 (0.362–1.175)	0.155		
Hypertension	1.071 (0.381–3.014)	0.896		
Neutrophil	1.159 (1.069–1.255)	< 0.001^*^	1.073 (0.991–1.163)	0.081
Monocytes	0.945 (0.711–1.256)	0.697		
Lymphocytes	0.795 (0.573–1.105)	0.172		
FARP	1.558 (1.299–1.868)	< 0.001^*^	1.416 (1.117–1.795)	0.004^*^
albumin	0.859(0.798–0.925)	< 0.001^*^		
fibrinogen	2.335 (1.510–3.612)	< 0.001^*^		
APTT	0.994 (0.954–1.035)	0.760		
TT	0.872 (0.741–1.025)	0.097^*^	1.122 (0.909–1.386)	0.283
D–Dimer	2.634 (1.308–5.303)	0.007^*^	1.505 (0.794–2.854)	0.210
CRP	1.031 (1.001–1.063)	0.044^*^	1.009 (0.984–1.034)	0.492

OR, Odds ratio; CI, confidence interval; FARP, fibrinogen albumin ratio percentage; APTT, activated partial thromboplastin time; TT, thrombin time; CRP, C-reactive protein.

^*^P < 0.05.

### 3.3. Correlation between FARP and anti-NMDAR encephalitis disease prognosis

Spearman's correlation analysis showed that the blood neutrophil count (*r* = 0.235, *P* = 0.001), Fib level (*r* = 0.271, *P* < 0.001), D-dimer level (*r* = 0.229, *P* = 0.002), and CRP level (*r* = 0.211, *P* = 0.010) were significantly correlated with the mRS score at discharge, but the correlation coefficients were low. FARP was a significant, strongly positive correlation with the final mRS (*r* = 0.312, *p* < 0.001). We observed no association of the blood lymphocyte count, blood monocyte count, APTT, TT, or Alb, with the final mRS (*P* > 0.05) (Table 5).

To investigate the factor potentially affecting disease prognosis, we performed a univariate logistic regression analysis, in which we found remarkable correlations of prognosis with the following variables: FARP (OR = 1.496, 95% CI = 1.245–1.797, *P* < 0.001), age at onset (OR = 1.024, 95% CI = 1.001–1.047, *P* = 0.039), blood neutrophil count (OR = 1.088, 95% CI = 1.014–1.167, *P* = 0.019), TT (OR = 0.794, 95% CI = 0.634–0.996, *P* = 0.046), and CRP (OR = 1.029, 95% CI = 1.004–1.054, *P* = 0.021). Moreover, we observed no significant relationship among sex, hypertension, blood monocyte count, blood lymphocyte count, APTT, or D-dimer level with the final mRS (*P* > 0.1). The multivariate model showed that FARP was an independent risk factor for disease prognosis (OR = 1.252, 95% CI = 1.010–1.552, *P* = 0.040) (Table 7).

**Table 7:** Binary logistic regression of disease prognosis in patients with anti-NMDAR encephalitis.

	**Univariable analysis**		**Multivariable analysis**	
	**OR (95%CI)**	***P***	**OR (95%CI)**	***P***
Age at onset	1.024 (1.001–1.047)	0.039^*^	1.013 (0.986–1.041)	0.356
Gander, female	0.827 (0.410–1.706)	0.607		
Hypertension	0.750 (0.227–2.476)	0.637		
Neutrophil	1.088 (1.014–1.167)	0.019^*^	1.061 (0.997–1.129)	0.064
Monocytes	1.097 (0.818–1.471)	0.538		
Lymphocytes	1.034 (0.923–1.157)	0.566		
FARP	1.496 (1.245–1.797)	< 0.001^*^	1.252 (1.010–1.552)	0.040^*^
Albumin	0.924 (0.853–1.001)	0.053^*^		
Fibrinogen	2.587 (1.603–4.175)	< 0.001^*^		
APTT	0.980 (0.923–1.040)	0.502		
TT	0.794 (0.634–0.996)	0.046^*^	0.989 (0.772–1.267)	0.932
D–Dimer	1.054 (0.886–1.254)	0.553		
CRP	1.029 (1.004–1.054)	0.021^*^	1.016 (0.993–1.039)	0.172

OR, Odds ratio; CI, confidence interval; FARP, fibrinogen albumin ratio percentage; APTT, activated partial thromboplastin time; TT, thrombin time; CRP, C-reactive protein.

^*^P < 0.05.

### 3.4. ROC curve for FARP predicts disease severity and prognosis in patients with anti-NMDAR encephalitis

**Figure 2:**
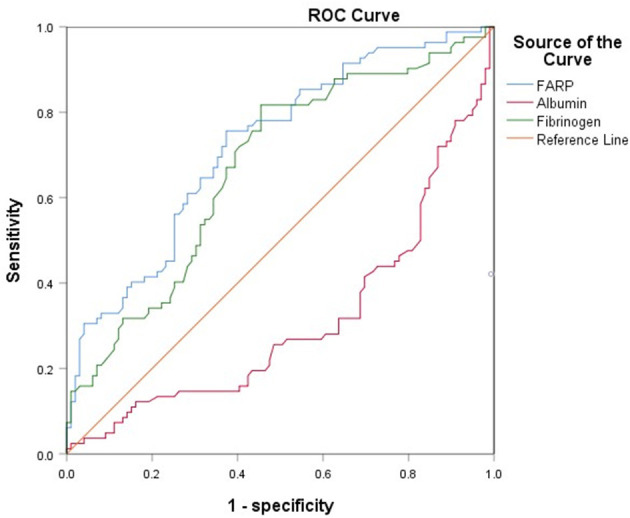
The ROC curve of FARP predicts the severity of anti-NMDAR encephalitis.

**Figure 3:**
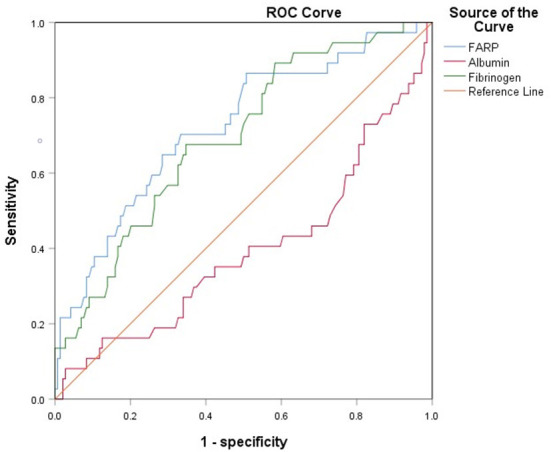
The ROC curve of FARP predicts the prognosis of anti-NMDAR encephalitis.

The ROC curve for FARP at admission (Figure 2) was used to predict disease severity, and the ROC curve for FARP at discharge (Figure 3) was used to predict prognosis. For predicting disease severity, the area under the ROC curve was 0.722 (95% CI 0.648–0.796), the optimal cutoff value was 6.50%, the sensitivity was 75.6%, and the specificity was 62.6% (Table 8). For predicting disease prognosis, the area under the ROC curve was 0.723 (95% CI 0.629–0.817), the optimal cutoff value was 7.22%, the sensitivity was 70.3%, and the specificity was 66.7% (Table 9).

**Table 8:** ROC curve analysis of the prediction of disease severity based on FARP.

**Item**	**Cutoff value**	**Sensitivity**	**Specificity**	**AUC**	**95% CI**	***P*-value**
FARP	6.4950	75.6%	62.6%	0.722	0.648–0.796	< 0.001^*^
Fibrinogen	2.6450	81.7%	54.5%	0.671	0.592–0.750	< 0.001^*^

AUC, area under curve; CI, confidence interval; FARP, fibrinogen albumin ratio percentage.

^*^p < 0.05.

**Table 9:** ROC curve analysis of the prediction of disease prognosis based on FARP.

**Item**	**Cutoff value**	**Sensitivity**	**Specificity**	**AUC**	**95% CI**	***P*-value**
FARP	7.2150	70.3%	66.7%	0.723	0.629–0.817	< 0.001^*^
Fibrinogen	2.9950	67.6%	65.3%	0.694	0.602–0.786	< 0.001^*^

AUC, area under curve; CI, confidence interval; FARP, fibrinogen albumin ratio percentage.

^*^p < 0.05.

## 4. Discussion

**Figure 4:**
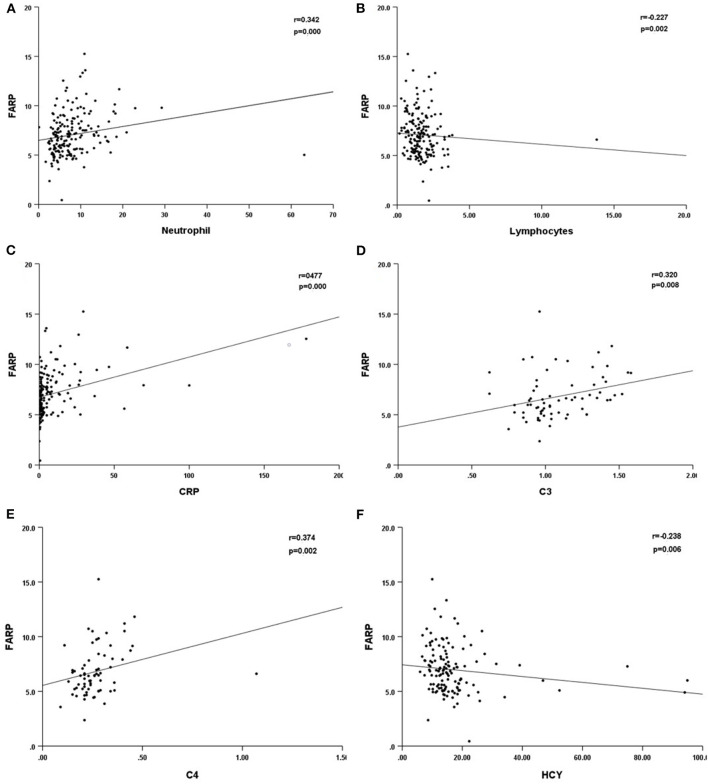
Scatter plot of the correlation between neutrophil (^*^10^9^/L) **(A)**, lymphocytes (^*^10^9^/L) **(B)**, CRP (mg/L) **(C)**, C3 **(D)**, C4 **(E)**, HCY **(F)** and FARP level. CRP, C-reactional protein; C3, complement 3; C4, complement 4; HCY, homocysteine.

Previous studies have demonstrated the important role of FARP, Fib, and albumin in immune-related diseases. In this research, we compared the differences in the routine blood test and coagulation function between healthy people and patients with anti-NMDAR encephalitis at admission and discharge. We found that high FARP and Fib levels were related to the more serious diseases at onset and discharge, while low albumin level was only related to the more serious diseases at onset. FARP was found to be an independent predictor of the severity and prognosis of anti-NMDAR encephalitis. In addition, FARP level in patients with anti-NMDAR encephalitis was positively correlated with neutrophils, CRP, C3, and C4 levels and negatively correlated with lymphocyte count and serum homocysteine (HCY) levels (Table 10, Figure 4).

**Table 10:** Correlation between FARP and other laboratory indicators.

	***r***	***p***
Neutrophil	0.342	< 0.001^*^
Lymphocytes	−0.227	0.002^*^
CRP	0.477	< 0.001^*^
C3	0.320	0.008^*^
C4	0.374	0.002^*^
HCY	−0.238	0.006^*^

r, relevance; CRP, C-reactional protein; C3, complement 3; C4, complement 4; HCY, homocysteine.

^*^P < 0.05.

According to the results of stratification based on mRS score at admission and discharge, the serum Alb level in the severe group was significantly lower than that in the mild-to-moderate group, which was consistent with the previous research (19). In the correlation analysis, the serum Alb level was strongly correlated with the severity of neurological impairment in anti-NMDAR encephalitis but did not correlate with the short-term prognosis of the disease. It has been reported that in autoimmune diseases, a low Alb level before treatment is an important indicator of prognosis in the short and long term (16). Serum Alb is a multifunctional plasma protein that downregulates the inflammatory cascade and is largely responsible for antioxidant capacity, which reduces the underlying inflammatory processes in patients with autoimmune disorders. Alb is the main serum protein involved in drug binding and transport. Many primary medications used to treat autoimmune diseases, including prednisolone, methylprednisolone, and non-steroidal immunosuppressant mycophenolate mofetil, are bound to proteins after administration. Their efficacy and safety may be affected by any reduction in the availability of binding sites on Alb, thus affecting treatment effectivity (20). Neubert et al. found that Alb can inhibit the formation of neutrophil extracellular traps (NETosis) by scavenging activators such as lipopolysaccharide (LPS), which is a key to an immune defense mechanism involved in the pathogenesis of autoimmune, inflammatory, and neoplastic diseases (21). Low Alb levels are associated with a variety of autoimmune and inflammatory diseases of the CNS. Studies have indicated that low levels of Alb are independent parameters for exacerbation of severe neurological impairment in patients in the acute phase of neuromyelitis optica spectrum disorders (NMOSD). Other studies have shown that serum Alb levels are inversely related to serum IL-33 concentration during the acute stage of NMOSD, indicating that serum Alb may be involved in the immunopathological process of the disease partly *via* the interaction with IL-33 (22). Although Alb levels showed a strong correlation with disease severity, the result of the area under the ROC curve indicates that it still lacks evidence as a predictor of anti-NMDAR encephalitis disease severity.

Fib, a glycoprotein synthesized and secreted by hepatocytes and circulating into the blood, is an important component of the coagulation and hemostasis system (23). It can simultaneously play significant roles in coagulation, inflammation, and tissue repair (9, 24). Moreover, as a blood coagulation protein, Fib plays an important role in CNS diseases. Many diseases of the nervous system are associated with systemic inflammation. Inflammation itself results in increased blood Fib content (25). In traumatic injury, neurodegenerative and inflammatory diseases, a major change in the molecular composition of the extracellular microenvironment is caused by a large infiltration of plasma Fib into the CNS through the damaged BBB, which is transformed into insoluble Fib through tissue factors and thromboplastic proteins that are abundant after damage (26, 27). In multiple sclerosis (MS) and other diseases, encephalitogenic adaptive immune responses are induced by Fib, which recruits peripheral macrophages into the CNS, leading to demyelination (28). In an experimental encephalomyelitis mouse model, Fib in extracellular vesicles, driven by CD8+ cells, induced a spontaneous relapsing-remitting disease phenotype, indicating that Fib contributes to the persistence of neuroinflammation and disease recurrence (29). In addition, the study found that fibrinogen, as a kind of coagulant protein deposited in CNS after BBB destruction, induced intracranial adaptive immune response, and peripheral macrophages were recruited into CNS, leading to demyelination. This indicated that the final product of the coagulation cascade was the key factor of CNS autoimmunity (28). The molecular structure of Fib contains binding sites for receptors expressed by cells of the nervous system and proteins that regulate key functions. Its pro-inflammatory functions are mediated predominantly through binding to CD11b/CD18 integrin receptors (also known as αMβ2 or complement receptor 3) in microglia and macrophages, leading to functional defects in neuroinflammatory diseases (26). In addition, previous studies have reported that Fib is significantly correlated with interleukin (IL)-6 in ankylosing spondylitis (30). After brain trauma, it was found that compared with serum, IL-6 in cerebrospinal fluid increased 40–100 times, and the levels of some acute-phase proteins, including Fib, increased accordingly, triggering a positive feedback loop, which exacerbated the inflammatory reaction by increasing IL-6 levels (25). High Fib levels aggravate inflammatory, autoimmune, and neoplastic diseases (31–33). Zhang et al. reported that patients with anti-NMDAR encephalitis and high Fib levels obtained poor effects after first-line treatment (14). However, there have been few reports on the predictive role of Fib in the severity and prognosis of anti-NMDAR encephalitis. Our study clarified that Fib levels were significantly increased in the severe group of patients with anti-NMDAR encephalitis at admission and discharge. Moreover, Fib level was related to disease severity and prognosis in the correlation analysis.

FARP is the percentage of Fib divided by that of Alb. Fib and Alb are two critical factors involved in inflammation, nutrition, and coagulation. The FARP has been extensively studied in tumors, cardiovascular, rheumatic immune, and other diseases (33–38). Wang et al. (39) indicated that preoperative evaluation of FARP has prognostic value for patients undergoing hepatectomy for colorectal liver metastases. A retrospective study showed that the FARP level was independently associated with the presence and severity of disease in patients with acute coronary syndrome (34). Hizli et al. found that high levels of FARP may predict inflammation in patients with moderate-to-severe sleep apnea syndrome (40). In addition, other researchers have indicated that a high FARP level is independently associated with the risk of hemorrhagic transformation after acute cerebral ischemia (41). In patients with MS relapses, FARP levels were significantly higher, which could place this factor as a useful indicator of relapses (32). However, few studies have assessed the role of FARP in autoimmune diseases of the CNS. In this regard, our study found, for the first time, a relationship between FARP levels and anti-NMDAR encephalitis severity and prognosis. Indeed, FARP levels in the group classified as severe based on mRS scores at admission and discharge were dramatically higher than that in the mild-to-moderate group and were significantly associated with disease severity and prognosis.

In anti-NMDAR encephalitis, Th17 cells are accumulated in CSF. Also, the concentration of cytokines such as IL-17 is remarkably increased. Furthermore, IL-17 significantly correlates with the prognosis and relapse likelihood of anti-NMDAR encephalitis (42). FARP is negatively correlated with Th17 cell numbers and IL-17A levels in the serum of patients with rheumatoid arthritis (43). These studies indicate that FARP is probably a potential marker of anti-NMDAR encephalitis. The C4 inflammation indicator is reportedly associated with the severity of anti-NMDAR encephalitis (44). Our research found that CRP was related to disease severity and short-time prognosis of anti-NMDAR encephalitis, and CRP, C3, and C4 were significantly correlated with FARP (Table 10). Thus, further studies add to evidence showing that FARP plays an important role in the inflammatory process in anti-NMDAR encephalitis.

In addition, it has been discovered that the FARP level in our patients was negatively correlated with HCY. Liu et al. demonstrated that the changes in HCY level in patients with anti-NMDAR encephalitis were negatively correlated with changes in the mRS score at 3-month follow-up. They concluded that the high HCY level leads to disbalance in the immunity system, reducing the protection of the immune system against inflammatory reactions and neurotoxicity in anti-NMDAR encephalitis (45). This further illustrated the key role of FARP in the autoimmune inflammatory response of anti-NMDAR encephalitis. Thus far, the potential mechanism by which FARP supports anti-NMDAR encephalitis remains unclear.

Here, to the best of our knowledge, we first determined the predictive role of FARP in anti-NMDAR encephalitis. Nevertheless, a few deficiencies were found in this research. First, this retrospective study lacks data on the late follow-up status of patients and cannot be used to elucidate the causation between FARP and anti-NMDAR encephalitis disease activity. Second, FARP cannot fully represent the inflammatory state of anti-NMDAR encephalitis. Therefore, before using FARP as a clinical biomarker of anti-NMDAR encephalitis, a large amount of data are needed to conduct multicenter and long-term studies.

## 5. Conclusion

In brief, this research showed that FARP level is enhanced in patients with anti-NMDAR encephalitis and is significantly positively related to anti-NMDAR encephalitis disease severity and prognosis. This new indicator could be used as a useful predictor of the severity and prognosis of patients with anti-NMDAR encephalitis. The determination of FARP is simple, convenient, rapid, and relatively non-traumatic. More aggressive therapies should be given when these predictors are observed. In this regard, it is necessary to elucidate the potential mechanisms of FARP in anti-NMDAR encephalitis pathogenesis and further validate these conclusions.

## Data availability statement

The original contributions presented in the study are included in the article/supplementary material, further inquiries can be directed to the corresponding author.

## Ethics statement

The studies involving human participants were reviewed and approved by the Ethics Committee of Zhengzhou University (2019-KY-018). The patients/participants provided their written informed consent to participate in this study.

## Author contributions

JD contributed to the design of the research, performed data analysis, and wrote the original manuscript. JD, YSh, YSo, KW, and XY completed the data collection and curation. ZG, YL, YY, and YJ contributed to conception and the revising of the manuscript. All authors contributed to the article and approved the submitted version.
